# Real-Time fMRI Neurofeedback Training as a Neurorehabilitation Approach on Depressive Disorders: A Systematic Review of Randomized Control Trials

**DOI:** 10.3390/jcm11236909

**Published:** 2022-11-23

**Authors:** Pamela P. González Méndez, Julio Rodino Climent, Jeffrey A. Stanley, Ranganatha Sitaram

**Affiliations:** 1 Department of Psychiatry, School of Medicine, Pontificia Universidad Católica de Chile, Santiago 8331150, Chile; 2 Brain Dynamics Laboratory, School of Biomedical Engineering, Universidad de Valparaíso, Valparaíso 2362905, Chile; 3 Department of Psychiatry and Behavioral Neurosciences, School of Medicine, Wayne State University, Detroit, MI 48202, USA; 4 Diagnostic Imaging Department, St. Jude Children’s Research Hospital, Memphis, TN 38105, USA

**Keywords:** neuromodulation, neurofeedback, self-regulation, psychiatry disorders, depression, real-time fMRI, RCT, systematic review

## Abstract

Real-time functional magnetic resonance imaging neurofeedback (rt-fMRI-nf) training is an emerging intervention for neurorehabilitation. However, its translation into clinical use on participants with clinical depression is unclear, the effect estimates from randomized control trials and the certainty of the supporting evidence on the effect estimates are unknown. As the number of studies on neurofeedback increases every year, and better quality evidence becomes available, we evaluate the evidence of all randomized control trials available on the clinical application of rt-fMRI-nf training on participants with clinical depression. We performed electronic searches in Pubmed, Embase, CENTRAL, rtFIN database, Epistemonikos, trial registers, reference lists, other systematic reviews, conference abstracts, and cross-citation in Google Scholar. Reviewers independently selected studies, extracted data and evaluated the risk of bias. The certainty of the evidence was judged using the GRADE framework. This review complies with PRISMA guidelines and was submitted to PROSPERO registration. We found 435 results. After the selection process, we included 11 reports corresponding to four RCTs. The effect of rt-fMRI-nf on improving the severity of clinical depression scores demonstrated a tendency to favor the intervention; however, the general effect was not significant. At end of treatment, SMD (standardized mean difference): −0.32 (95% CI −0.73 to 0.10). At follow-up, SMD: −0.33 (95% CI −0.91, 1.25). All the studies showed changes in BOLD fMRI activation after training; however, only one study confirmed regulation success during a transfer run. Whole-brain analyses suggests that rt-fMRI nf may alter activity patterns in brain networks. More studies are needed to evaluate quality of life, acceptability, adverse effects, cognitive tasks, and physiology measures. We conclude that the current evidence on the effect of rt-fMRI-nf training for decision-making outcomes in patients with clinical depression is still based on low certainty of the evidence.

## 1. Introduction

The magnitude of depressive disorders’ symptoms in terms of public health is enormous. Global prevalence of clinical depression is estimated around 322 million people, increasing 18.4% between 2005 and 2015 [1]. In addition, depression is ranked by the WHO as the single largest contributor to global disability (7.5% of all years lived with disability in 2015) [1]. Pharmacological and non-pharmacological treatments are available; however, there is still a need to investigate and develop novel interventions that could enhance treatments effectiveness, account for lower adverse effects, or target resistance-to-treatment populations. 

In this regard, Brain-Computer Interfaces-based neurofeedback is an innovative neuromodulation therapy that is considered a new field of research. Neurofeedback is a type of biofeedback in which neural activity is measured, and a visual, an auditory or another representation of brain activity is presented to the subject in real time to facilitate self-regulation of the putative neural substrates that underlie a specific behavior or pathology [2]. Neurofeedback helps to volitionally regulate brain activity through training the subject [2,3]. It began with experiments showing that humans could volitionally manipulate electroencephalographic signals in real time [4]. Up to date, non-invasive neurofeedback training has been built on different modalities, including electroencephalography (EEG), near-infrared spectroscopy (NIRS), magnetoencephalography (MEG), functional magnetic resonance imaging (fMRI), or a combination of modalities. In this systematic review, we focus on real-time neurofeedback training based on fMRI modality, which is called real-time functional magnetic resonance imaging neurofeedback (rt-fMRI-nf). 

The main strengths of using fMRI for neurofeedback training compared with other modalities include the high spatial resolution, reasonable temporal resolution of the order of seconds, the ability to cover the whole brain, including deeper brain areas [5,6], and the safety of this technique regarding adverse effects [7]. Functional MRI is based on Blood Oxygen Level Dependent signals [8] and its hemodynamic response function which represents an indirect measure that correlates with neural activity [9,10,11]. Real time refers to the process in which functional data has been analyzed and displayed simultaneously with data acquisition. Thus, a BCI (Brain–Computer Interface) system based on real-time fMRI allows subjects the possibility to observe in real time their own BOLD (Blood Oxygen Level Dependent) signal response and volitionally modulate it through mental learning techniques across sessions, with potential in neurorehabilitation [2,12,13], mainly throughout the normalization of altered neuroimaging patterns found in the disorder.

Real-time fMRI neurofeedback training can regulate brain activity or specific networks and in consequence behavior; however, it is still in an early stage, and it remains unclear if those brain and behavioral changes can translate into clinical relevance [14,15,16]. However, as the number of published studies is increasing every year and better quality study designs are available, we aim to investigate the current rt-fMRI-nf training evidence. Until now, similar systematic reviews [14,17], had opted for a qualitative synthesis instead of a meta-analysis approach, providing a description about a variety of experimental design, different control conditions, different modalities for neurofeedback, and focused on quality reporting to provide recommendations. Thus, as an alternative approach, we include a quantitative synthesis (meta-analysis), for which we decided to prospectively reduce heterogeneity by narrowing our clinical question to clinical efficacy, gathering only rt-fMRI-nf modality, participants with a depressive disorder, and only randomized control trials as the most appropriate designs to address the benefits of an intervention. Thus, providing an estimated combined effect of rt-fMRI-nf training on severity depression scores in combination with the assessment of the certainty of the current evidence in which the estimate effect is based on.

To our understanding, this is the first systematic review to include a GRADE (Grading of Recommendations, Assessment, Development and Evaluations) framework of the certainty of the evidence, and therefore a Summary of Findings (SoF) table that is presented as a summary that conveys the results in a straightforward style for health decision makers (including physicians, informed consumers, and health policy makers). Additionally, regarding this clinical question, we are the first systematic review that pre-registered its protocol as a quality statement. 

Our contribution aims to promote evidence-based clinical decision-making, and to provide recommendations for future neuroscience research on fMRI-based neurofeedback training in patients with clinical depression.

## 2. Methods

This manuscript complies with the ‘Preferred Reporting Items for Systematic reviews and Meta-Analyses’ (PRISMA) guidelines for reporting systematic reviews and meta-analyses [18]. The PRISMA statement consists of a 27-item checklist and a four-phase flow diagram to help authors improve the reporting of systematic reviews and meta-analyses. The review was registered in PROSPERO CRD42020192096, and the protocol was published and is available on medRxiv MEDRXIV/2020/159319. We built our research question using the PICOS (Population, Intervention, Comparison, Outcome, Study design) framework first introduced in 1995 [19], the PICO framework is recommended to address clinical questions such as the one addressed in this Systematic Review and helps to improve database searching [20,21].

### 2.1. Information Sources 

We conducted electronic searches for randomized controlled trials, without publication status or language restrictions in Pubmed, Embase and CENTRAL. We searched in Epistemonikos database for reviews that might contain related trials. We searched for trial registers that we found through the database search, we also searched manually in trial registers, such as ClinicalTrials.gov and the International Clinical Trials Registry Platform (ICRTP) for additional reports. To identify articles that might have been missed in the electronic searches, we also reviewed the reference lists of all included studies, and other systematic reviews for additional potentially eligible primary studies. We also searched in rtFIN database “http://www.rtfin.org/literature.html (accessed on 27 July 2020)”, reviewed rtFIN conference abstracts, Human Brain Mapping (OHBM) conference abstracts, Society of Neuroscience (sfn) conference abstracts, and conducted a cross-citation search in Google Scholar using each included study as the index reference.

### 2.2. Search Strategy 

The search strategy was developed with the help of a librarian experienced in searching systematic reviews, using the combination of terminologies describing the intervention and the target population. The searches were performed in June 2020 and updated in June 2021. The syntax of the search was adapted for each database, and they can be found in the supplementary material.

### 2.3. Eligibility Criteria

During the selection process, we consider the population, intervention, comparison, and study design components as inclusion criteria. We did not consider outcomes as inclusion criteria during the selection procedure. Reports meeting all the criteria except for the outcome criterion were preliminarily included and evaluated in full text. We applied no language restrictions.

#### 2.3.1. Population

We included trials assessing participants clinically diagnosed with a depressive disorder. We defined clinical depression according to the conditions described on the ‘Diagnostic and Statistical Manual of Mental Disorders—Fifth Edition (DSM-V criteria) [22]. Common features of these disorders are the presence of sad, empty, or irritable mood, accompanied by somatic and cognitive changes that significantly affect the individual’s capacity to function. What differs among them are issues of duration, timing, or presumed etiology [22]. Thus, according to DSM-V we considered as inclusion criteria depressive disorders such as major depressive disorder, including major depressive episode, persistent depressive disorder (dysthymia), premenstrual dysphoric disorder, other specified depressive disorder, and unspecified depressive disorder, as defined by authors on their primary studies. To avoid confounding effects especially regarding overlaps in the region of interest when there are multiple disorders or different substance/medications involved, we excluded the substance/medication-induced depressive disorder, depressive disorder due to another medical condition, and disruptive mood dysregulation disorder, which was a new diagnosis included in DSM-V for children up to age 12 years who exhibit persistent irritability and frequent episodes of extreme behavioral dyscontrol, a symptom pattern that typically develops into unipolar depressive disorders or anxiety disorders. Additionally, it is important to acknowledge that most of the participants are not medication-free nor comorbidity-free (see Table 1 and Table S1 for more information of the population characteristics on the included studies).

Note: the population that we found also included comorbid psychiatric disorders as secondary condition, which is common. We judge to include the studies if they directly assessed the disorders in our inclusion criteria, as the primary diagnosis. 

#### 2.3.2. Intervention

The intervention of interest is real-time functional MRI neurofeedback (rt-fMRI-nf) training. We did not limit our criteria to any number, frequency, or length of neurofeedback sessions. We did not restrict any feedback display (e.g., auditory, visual, tactile, proprioceptive) or feedback format (e.g., simple graphic, melody, tone, video clip). We did not restrict how feedback was given (e.g., number of feedback sessions and runs, number of trainings, feedback strategy, feedback modality and content, amount of reward, strength of the magnetic field).

#### 2.3.3. Comparison 

The comparison of interest is placebo (e.g., sham neurofeedback, neurofeedback from a largely unrelated brain region, inversing the neurofeedback reward contingency), another active non-neurofeedback control (e.g., any similar type of computerized cognitive training, medication, or standard therapy) or no treatment (i.e., neurofeedback + standard treatment vs. standard treatment alone).

#### 2.3.4. Outcome 

We included the following outcomes according to recommendations by the Consensus on the reporting and experimental design of clinical and cognitive-behavioral neurofeedback studies (CRED-nf) created for neurofeedback [23], and recommendations from the Research Domain Criteria (RDoC) project proposed by the National Institute of mental Health (NIMH) made for neuroscience research [24]. Our primary outcome measure was symptomatology/disease severity reduction. Secondary outcomes were quality of life, acceptability, and adverse effects. Primary and secondary outcomes are presented at the Summary of Findings table (SoFt). Other outcomes were defined as follows: 1. brain MRI metrics (e.g., BOLD signal percentage changes and individual differences on upregulation and downregulation, DTI changes, cortical thickness changes); 2. cognitive tasks (i.e., a working memory task); 3. physiology measures defined as well-established indices of certain constructs, but that do not necessarily tap the studied system directly, other than BOLD activation of cortical regions (e.g., heart rate, breathing, average cortisol levels, PET, NIRS).

#### 2.3.5. Study Design

We only included randomized control trials. We excluded studies evaluating the effects on animal models.

### 2.4. Selection of the Studies Process 

The results of the literature search were incorporated into the screening software CollaboratronTM [25]. Two researchers (PGM, JRC) independently screened the titles and abstracts yielded by the search against the eligibility criteria. We obtained the full reports for all titles that appeared to meet the eligibility criteria or required further analysis to decide on their inclusion. When there were doubts about one or more eligibility criteria, we contacted the authors by e-mail to resolve inclusion. Discrepancies between the review authors were solved by discussion to reach consensus. When necessary, a third review author was consulted to achieve a decision. We recorded the reasons for excluding trials in any stage of the search and outlined the study selection process in a PRISMA flow diagram adapted for the purpose of this research.

### 2.5. Data Collection Process

Two reviewers independently conducted data extraction from each included study using the standardized methodology proposed by the Cochrane Collaboration Group. Thus, we constructed an Excel document based on the Template for Intervention Description and Replication (TIDieR) [26] which provides a comprehensive framework for the full description of interventions and has been proposed for use in systematic reviews as well as reports of primary studies [27]. We collected information regarding: study design; participant characteristics, including study eligibility criteria, diagnostic criteria, characteristics of participants at the beginning of the study (e.g., age, sex, comorbidity and disease severity); the intervention, including description of the intervention(s) and comparison intervention(s), ideally with sufficient detail for replication (e.g., data acquisition parameters, neurofeedback protocol, signal processing, training characteristics); outcomes, including measurement tool or instrument (i.e., rating scales’ characteristics), specific metric used (e.g., percentage changes from baseline to a post-intervention time point, or post-intervention presence yes/no), method of aggregation (e.g., mean and standard deviation of scores in each group and timing of outcome measurements); results; source of funding; conflicts of interest disclosed by the researchers and risk of bias assessment for each individual study. Discrepancies between review authors were resolved by discussion to reach consensus. When necessary, a third review author was consulted to achieve a decision. When information was unclear, we contacted the study authors by e-mail to confirm the data.

### 2.6. Study Risk of Bias Assessment 

The Cochrane Collaboration group define bias as a systematic error, or deviation from the truth in results [28]. Bias may be distinguished from quality, because a study may be performed to the highest possible standards yet still have an important risk of bias. For example, in many situations it is impractical or impossible to blind participants or study personnel. Then, it is unfortunate to describe all such studies as of low quality, but that does not mean they are free of bias resulting from knowledge of intervention. Then, we emphasize that the risk of bias assessment should not be mistaken as a quality assessment tool. The tool focused on a single concept: risk of bias. It did not consider other concepts such as the quality of reporting, precision (the extent to which results are free of random errors), or external validity (directness, applicability or generalizability) [28]. Imprecision (random error) is reflected in the confidence interval around the intervention effect estimate from each study and in the weight given to the results of each study in the meta-analysis. Generalizability assessment depends on the purpose for which the study is to be used. We assessed the risk of bias for each randomized trial using the first version of the Cochrane Collaboration tool for assessing risk of bias in randomized trials [29]. The overall risk of bias was judged according to primary and secondary outcomes of our systematic review. Overall bias of each outcome studied was determined according to the following six domains: selection bias (systematic differences between baseline characteristics of the groups that are compared; evaluated through random sequence generation and allocation concealment), performance bias (systematic differences between groups in the care that is provided, or in exposure to factors other than the interventions of interest; evaluated through blinding of participants and personnel), detection bias (systematic differences between groups in how outcomes are determined; evaluated through blinding of outcome assessment), attrition bias (systematic differences between groups in withdrawals from a study; evaluated through incomplete outcome data), reporting bias (systematic differences between reported and unreported findings; evaluated through selective outcome reporting), and other bias (any important concerns about bias that are not addressed in the other domains of the tool). The risk of bias was evaluated as ‘low risk of bias’, ‘unclear risk of bias’ or ‘high risk of bias’ for each of these six domains. We prepared risk of bias tables explaining and justifying the allocation of the risk of bias (see Table S2a–d). Discrepancies between review authors were resolved by discussion to reach consensus. When necessary, a third review author was consulted to achieve a decision.

### 2.7. Effect Measures

For dichotomous outcomes, if available, we express the estimate of treatment effect of an intervention as risk ratios (RR) or odds ratios (OR) along with 95% confidence intervals (CI). For continuous outcomes, if available, we use mean difference (MD) and standard deviation (SD) to summarize the data, using a 95% CI. For continuous outcomes reported using different scales, the treatment effect is expressed as a standardized mean difference (SMD) with a 95% confidence interval. 

### 2.8. Synthesis Methods 

The results of the search and the selection of the studies are presented in the corresponding flow chart, according to recommendations of the PRISMA statement [18]. For outcomes where data was insufficient to calculate an effect estimate, a narrative synthesis is presented. For any outcome where data were available from more than one trial, we conducted a formal quantitative synthesis (meta-analysis) for studies clinically homogeneous using RevMan5 [30] and using the inverse variance method with the random-effects model. We assessed inconsistency mainly by visual inspection of the forest plots and using the I^2^ index.

### 2.9. Certainty Assessment

The certainty of the evidence for primary and secondary outcomes was judged using the Grading of Recommendations Assessment, Development and Evaluation working group methodology (GRADE Working Group) [31], across the domains of risk of bias, inconsistency, indirectness, and imprecision. For the main comparisons of primary and secondary outcomes, we prepared a Summary of Findings (SoF) table [32] using GRADEpro GDT software “https://gradepro.org (accessed on 9 November 2021)”.

## 3. Results

### 3.1. Study Selection

**Figure 1:**
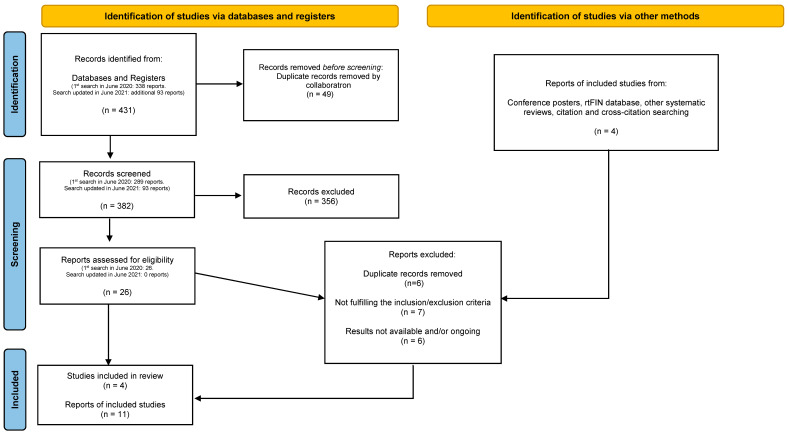
PRISMA flow diagram.

A total of 435 reports were identified through databases, registers, and other sources in June 2020. Later, we updated the databases search in June 2021, in which we identified and screened 93 additional reports; however, no extra reports that fulfilled inclusion criteria were found in this new search. The results of the literature searches were incorporated into the screening software ‘CollaboratronTM’ [25] where 49 duplicate records were automatically removed. After duplicates removal and the screening process against the inclusion/exclusion criteria, when inclusion criteria and/or information were unclear, we contacted study authors by e-mail to confirm data. The response rate was 67%; we finally excluded 356 records primarily due to not fulfilling the inclusion/exclusion criteria. Later, we reviewed the remaining 26 records in full text and excluded 17 reports. Additionally, we reviewed other sources for included studies and selected four additional reports. Finally, we included a total of four studies comprised of 11 reports that correspond to journal articles, a pre-print article, trial registries, and poster/abstract conferences. The study selection process is explained in a PRISMA flow diagram adapted for the purpose of this project, see Figure 1.

### 3.2. Description of the Included Studies

A summary of the characteristics of the included studies is provided on Table 1.

**Table 1:** Characteristics of studies.

Study ID	Population *				Intervention (rt-fMRI-NF)		Control			Number of Sessions		
Author (Year) (N° of Randomized Participants) Place of Recruitment	Type of Depressive Disorder (dg. Criteria) and Severity/Activity	Duration of the Disease, Mean (SD)	Age in Years, Mean (SD)	Include Participants Using Medication	NF Protocol	Mental Strategy	Type of Control	Control Protocol	Mental Strategy	NF	Transfer Run or Session	FU
Journal articles: Young et al. (2017a), Young et al. (2017b), Young et al. 2018) Poster presentation rt-FIN2019: “Structural equation model mapping for elucidating brain activity mediators of depression symptom reduction during real-time fMRI amygdala neurofeedback” Trial register: NTC02079610 (N = 36) Recruitment: U.S.A.	MDD (DSM-IV) Currently moderate	E: 30 (56) months C: 34 (49) months	Adults E: 32 (12) C: 31 (9)	No At the beginning of the study: average time since last antidepressant: 33 months. During the trial: Changes of dose not described	Visual NF ↑ from emotional processing brain area. Target ROI: L amygdala	Positive autobiographical memory recall	Neurofeedback from a largely unrelated brain signal	Visual NF ↑ from a brain area not involved in emotional processing. Target ROI: L HIPS	Positive autobiographical memory recall	2 training sessions 3 training runs per session	A transfer run each session	1 1 week after final treatment session
Journal article: Mehler et al. (2018) Trial register: NCT01544205 (N = 43) Recruitment: U.K.	Unipolar depression (MINI) Currently moderate or severe	E: 19 (12.39) years C: 18.56 (14.76) years	Adults E: 47 (13) C: 47 (13)	Yes At the beginning of the study: stable antidepressant medication, no change of dose in the preceding three months. During the trial: 3 patients in the NFE group increased their antidepressant medication, and 5 patients (4 in the NFE and 1 in the NFS group) decreased or stopped medication.	Visual NF ↑ from emotional processing brain areas. Target ROIs: limbic and frontal portions of the anterior cerebrum	Imagery of positive stimuli (However, patients were not restricted in their mental strategies and could use any strategy)	Neurofeedback from a largely unrelated brain signal	Visual NF ↑ from visual processing brain areas. Target ROIs: PPA as main target.	Imagery of scenes (However, patients were not restricted in their mental strategies and could use any strategy)	5 training sessions	1 transfer session (At session3)	1 6 weeks after final treatment session
Journal article: Zahn et al. (2019) Trial register: NCT01920490 (N = 31) Recruitment: Brazil	Past major depressive episode (MINI changing the assessment to life-time (“have you ever”) for DSM- IV, which was modified to allow for subtypes of depression to be assessed for past episodes (melancholic, atypical) In remission of symptoms for at least 2 months and known to have an increased vulnerability towards MDD	E+C: Past MDE for at least 2 months	Adults E: 45 (14) C: 45 (18)	Yes At the beginning of the study: stable antidepressant medication not described. During the trial: Changes of dose not described	Visual NF ↑ (during guilt condition) from brain connectivity areas previously identified as a signature of overgeneralised self-blaming emotions in MDD (self-blame-selective connectivity reductions). During the indignation condition, visual feedback reinforced stabilisation of the preceding degree of correlation between the aTL and sACC in both intervention groups. Target ROIs: Functional connectivity between aTL and sACC	Retrieving guilt and indignation/anger-related autobiographical memories	Changing the neurofeedback reward contingency	Visual NF → ← (during guilt condition) from brain connectivity areas previously identified as a signature of overgeneralised self-blaming emotions in MDD (self-blame-selective connectivity reductions). During the indignation condition, visual feedback reinforced stabilisation of the preceding degree of correlation between the aTL and sACC in both intervention groups. Target ROIs: Functional connectivity between aTL and sACC.	Retrieving guilt and indignation/anger-related autobiographical memories	1 training session	Not described	Not described
Pre-print article: Jaeckle et al. (2019) Trial register: ISRCTN10526888 (N = 43) Recruitment: U.K.	Early treatment-resistant MDD and recurrent MDD (DSM-5) In a current MDE, or partially remitted presenting with significantly impairing or bothering symptoms, rated three or four on the LIFE Interview.	E+C: minimum of one past MDE of at least a two-month duration. (Current MDE lasting > 12 months were excluded)	Adults E: 37 (11) C: 38 (10)	Yes At the beginning of the study: stable antidepressant medication, no change of dose in the preceding six weeks. During the trial: Changes of dose not described	Visual NF ↑ from brain connectivity areas related to self-blame vs. blaming others. Target ROIs: hyperconnected brain correlation patterns between the aTL seed and the posterior SC	Negative autobiographical memory recall (that would evoke strong feelings of self-blame and other blame, throughout strategies to manage their feelings of self-blame constructively). + use of a pre-defined list of psychological strategies (Participants could also develop their own strategies)	No NF treatment (Mental rehearsal alone)	Mental rehearsal outside the scanner.	Negative autobiographical memory recall (that would evoke strong feelings of self-blame and other blame, throughout strategies to manage their feelings of self-blame constructively). + use of a pre-defined list of psychological strategies (Participants could also develop their own strategies)	3 sessions 2 training runs per session	Not described	1 7–13 days after final treatment session)

Abbreviations: E: experimental group; C: control group; ROI: region of interest; Target ROI: region from which NF is calculated; L Amygdala = Left Amygdala; L HIPS = The left horizontal segment of the intraparietal sulcus; PPA = the parahipocampal place area; aTL = right superior anterior temporal lobe; sACC = subgenual cingulate cortex; posterior SC = posterior subgenual cortex; ↑ = upregulation; → ← = stabilization (reinforcing stabilization of the preceding degree of correlation between the aTL and sACC).; FU: follow-up; * See supplementary material for further details about exclusion criteria applied on the included studies and baseline characteristics of the population. Selected studies’ references: Young et al.: 2017 [33], 2017 [34], 2018 [35]; Mehler et al., 2018 [36]; Zahn et al., 2019 [37]; Jaeckle et al., 2019 [38], 2021 [39] (This trial was also published on a peer-review journal in 2021, posterior to the search date of this Systematic Review. Then, we did not count it as an additional report found through the search strategy. Regardless of that, the data extracted in this review from Jaeckle et al. did not change after publication).

We identified three main intervention components that vary across studies according to the design of the trial: region from which NF is calculated (target region), NF direction of regulation, and mental strategy. Regarding control groups, designs varied depending on what components of the intervention the authors intended to control for (see Table 2).

For the primary outcome, all selected studies used at least one of the following validated scales that measured severity of depression: MADRS, HDRS, BDI-II. Only one study assessed quality of life, none of the studies evaluate acceptability of the intervention, and three of the four selected studies assessed adverse effects. Regarding other outcomes, all selected trials reported brain MRI metrics. Only Young et al. reported cognitive tasks, and none of the trials reported physiology measures, other than BOLD activation (see Table 3).

### 3.3. Ongoing Studies and/or Results Not Available

The reports that were excluded because they were ongoing studies and/or its results were not available to be meta-analyzed are summarized in Table S3.

### 3.4. Risk of Bias in the Included Studies 

**Figure 2:**
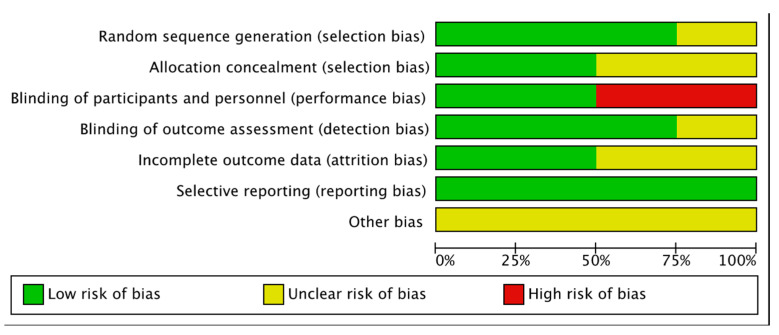
Risk of bias graph. Review authors’ judgements about each risk of bias item presented as percentages across all included studies.

**Figure 3:**
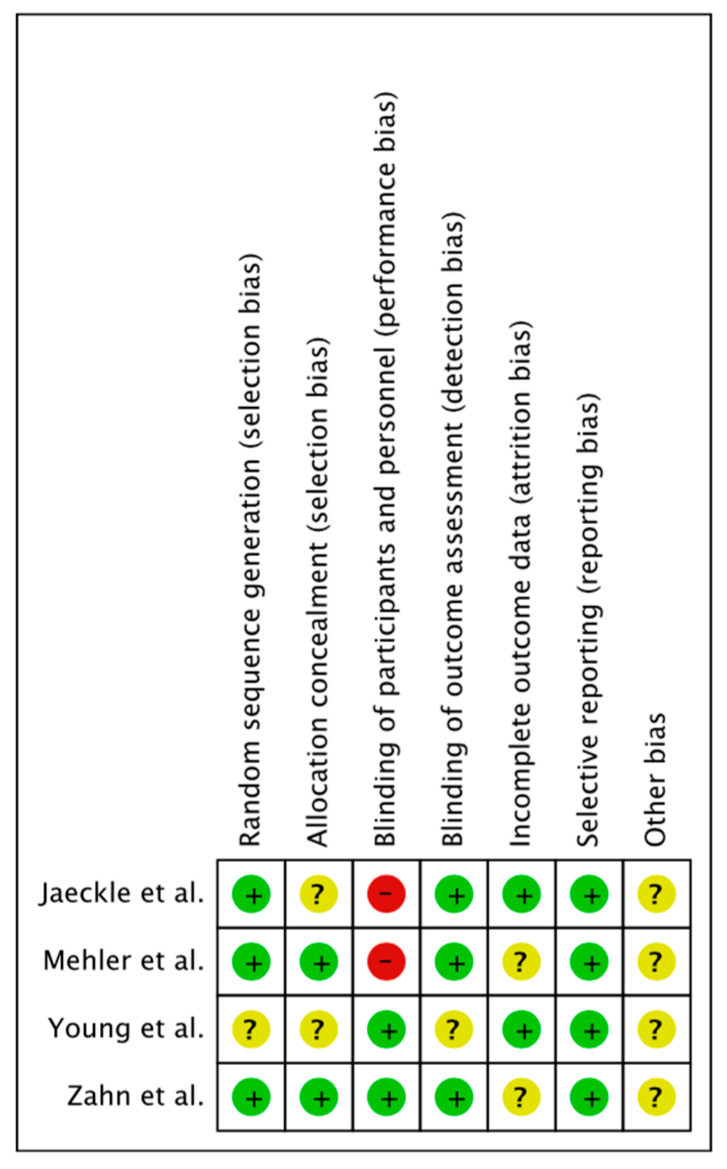
Risk of bias summary. Review authors’ judgements about each risk of bias item for each included study assessed by ROB−1 tool. Green (+): Low risk of bias; Yellow (?): Unclear risk of bias; Red (-): High risk of bias.

The overall risk of bias was assessed according to the primary and secondary outcomes of our systematic review. Most of the evaluated items had a low risk of bias. When insufficient information was provided to evaluate the risk of bias, the category was classified as unclear risk. Two studies were evaluated as high risk in performance bias. Overall risk of bias is presented as percentages across all included studies in Figure 2. A summary of the review authors’ judgements about each risk of bias item for each included study is provided in Figure 3. A summary table explaining the justifications for the allocation of the risk of bias is provided for each study in Table S2a–d. 

### 3.5. Certainty of the Evidence

Primary and secondary outcomes of this systematic review are defined as outcomes that are important to patients and other decision makers. For the main comparisons and primary and secondary outcomes, we prepared a Summary of Findings table (see Table 4). 

### 3.6. Primary Outcome 

#### 3.6.1. Symptomatology/Disease Severity Reduction at the End of Treatment

**Figure 4:**
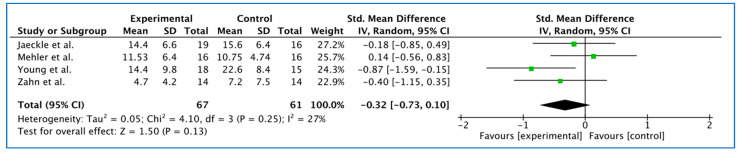
Forest plot. Reduction in depression severity clinical scores from baseline to the end of treatment. The figure displays for each study included in the meta-analysis the summary statistics (mean, standard deviation, and sample size) of depression severity clinical scores for the experimental and control groups, and its standard mean difference and its 95% confidence interval.

All studies reported this outcome at the end of treatment. The effect of rt-fMRI-nf on this outcome at the end of treatment is SMD: −0.32(−0.73, 0.10), with no important heterogeneity (I^2^ = 27%), presented in Figure 4; and with low certainty of evidence (see Table 4).

#### 3.6.2. Symptomatology/Disease Severity Reduction at Follow-Up

**Figure 5:**
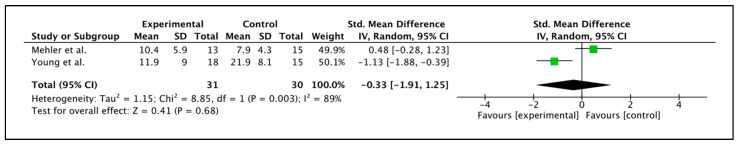
Forest plot. Difference in depression severity clinical scores from baseline to follow-up. The figure displays for each study included in the meta-analysis the summary statistics (mean, standard deviation, and sample size) of depression severity clinical scores for the experimental and control groups, and its standard mean difference and its 95% confidence interval.

Three studies mentioned follow-up measurement, but only two studies reported at this time point. One study reported a one-week follow-up period and the other a six-week follow-up period, both studies showed that changes in clinical symptoms persist after training; however, the follow-up periods reported are still in the range short-term periods. The effect of rt-fMRI-nf on this outcome at follow-up is SMD: −0.33(−0.91, 1.25), with significant heterogeneity (I^2^ = 89%), presented in Figure 5; and with very low certainty of evidence (see Table 4).

#### 3.6.3. Symptomatology/Disease Severity Reduction, Treatment Response

Two trials also reported treatment response rates, defined as an improvement ≥50% on depression severity scores. For this systematic review we considered participants who withdrew from the study or did not complete the trial as non-responders. The treatment response in Young et al. was 12/19 subjects in the experimental group versus only 2/17 in the control group. For Jaeckle et al. trial, 13/22 in the experimental group versus 12/21 in the control group. In Young et al. remission rates at the study’s end were found in six participants in experimental group versus one participant in the control group. Mehler et al., reported remission in four participants in experimental group versus eight participants in the control group. Zahn et al., only included remitted patients in the trial. No remission rates were reported on Jaeckle et al., authors of that study focused on reduce symptoms in MDD patients who have only insufficiently responded to standard treatment, rather than on remission rates.

#### 3.6.4. Other Symptomatology Measures

**Figure 6:**
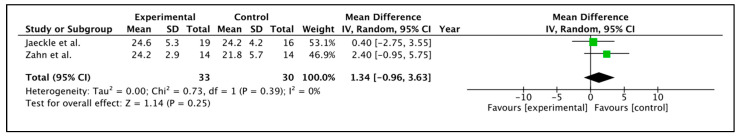
Forest plot. Increases self-esteem on the Rosenberg global self-esteem scale from baseline to the end of treatment. The figure displays for each study included in the meta-analysis the summary statistics (mean, standard deviation, and sample size) of depression severity clinical scores for the experimental and control groups, and its mean difference and its 95% confidence interval.

Two studies explored whether the intervention increases self-esteem using the Rosenberg global self-esteem scale, Jaeckle et al. used the 1965 version [40], and Zahn et al. used the 1989 version [41]. The effect of rt-fMRI-nf on this outcome at the end of treatment is MD: 1.34(−0.96, 3.63), with not important heterogeneity (I^2^ = 0%), presented in Figure 6; and with very low certainty of evidence (see Table 4).

### 3.7. Secondary Outcomes

#### 3.7.1. Quality of Life

Only one trial [36] reported quality of life assessment. The study used the 1982 Flanagan Quality of Life scale (QoLS) [42] and EuroQol research foundation questionnaire (EQ-5D-5L), which assessed the subject’s health utility. The Flanagan QoLS is an instrument to evaluate global quality of life. It is composed of 15 items covering five domains: physical and material well-being; relations with other people; social, community, and civic activities; personal development and fulfillment; and recreation. In each item, satisfaction, and importance of the item in the individual’s quality of life is evaluated. The EQ-5D-5L descriptive system comprises five dimensions: mobility, self-care, usual activities, pain/discomfort, and anxiety/depression. Each dimension has five levels: no problems, slight problems, moderate problems, severe problems, and extreme problems. The patient is asked to indicate his/her health state by ticking the box next to the most appropriate statement in each of the five dimensions. The five dimensions describes the patient’s health state. This trial reported no significant differences between groups in both scales.

#### 3.7.2. Acceptability

None of the selected trials reported acceptability outcomes.

#### 3.7.3. Adverse Effects

We analyzed data identified during the conduct of the review using an exploratory approach. An exploratory approach typically involves extracting any, or all, of the adverse event data found within the included studies [43]. We used the term ‘adverse event’ for an unfavorable or harmful outcome that occurs during, or after, the use of the intervention, but is not necessarily caused by it, and an adverse effect (or harm) as an adverse event for which the causal relation between the intervention and the event is at least a reasonable possibility [43]. In this systematic review, the analysis of rt-fMRI-nf adverse effects is limited, as we are only examining randomized control trials, also the method of reporting was not pre-specified and relies on spontaneous reporting. All of that, in addition with the complexity of the intervention, limit to establish the adverse effect causality. Then, we limited our summary to all the adverse events reported on the selected trials (see Table S4).

### 3.8. Other Outcomes

#### 3.8.1. Brain MRI Metrics, Changes in BOLD Activity—Regulation Success

The clinical feasibility of rt-fMRI-nf training depends on whether participants can continue to modulate their brain activity in the absence of feedback [14,44]. From the selected studies, only two studies described explicitly how they defined regulation success and included a transfer run or transfer session, among the two, only one suggest that participants can transfer their neural regulation to runs without neurofeedback. A description of regulation success outcomes is summarized in Table 5, and includes a definition of regulation success, regulation outcomes within and between groups, and related behavioral outcomes.

#### 3.8.2. Brain MRI Metrics, Changes in BOLD Activity—Whole Brain Analysis

From the selected trials, two reported whole-brain analyses (see Table 6).

#### 3.8.3. Cognitive Tasks

Only one study (Young et al.) assessed this outcome. The trial measured the increase in autobiographical memory test scores from baseline to follow-up. The results showed an increase in the percent of specific memories recalled and a decrease in the percent of overgeneral memories recalled in the experimental group.

#### 3.8.4. Physiology Measures

None of the selected trials reported physiology measures outcomes.

### 3.9. Recommendations

A secondary objective of our systematic review is to provide recommendations for future research. A recent published checklist [23] is available to encourage robust experimental design and clear reporting for clinical and cognitive-behavioral research. The checklist focuses mainly on aspects unique to the neurofeedback context that serves as a complement to the Consolidated Standards of Reporting Trials (CONSORT) guidelines. Because this checklist was published after that our selected trials, we did not evaluate the trials under this reporting standard. However, from the selected studies, poor descriptions regarding definition of regulation success and implementation of transfer runs or sessions, and lack of details from within sessions, limited the analysis of our systematic review. We encourage researchers to include in their research the recommendation of this neurofeedback checklist, which provides an online application available at “https://crednf.shinyapps.io/CREDnf (accessed on 11 November 2021)”, also we encourage researchers to evaluate regulation success and to explicitly state the definition of regulation success.

Additionally, to facilitate readers to evaluate how generalizable the findings of the randomized controlled trials are, we encourage researchers to provide more details on the population at recruitment level and to include educational level and socioeconomic status of the population and we encourage to state explicitly the presence or absence of changes of antidepressant dose or co-interventions not only at the beginning of the trial, but also during the trial, those were weakness of the available RCTs. 

Concerning the intervention description, we encourage the usage of the Template for Intervention Description and Replication (TIDieR) [26,27] which provides a comprehensive framework for full description of interventions and has been proposed for use in systematic reviews as well as reports of primary studies. The ‘TIDieR’ checklist can be found at “https://www.equator-network.org/reporting-guidelines/tidier (accessed on 11 November 2021”. The selected studies provide sufficient information regarding the rational of the elements essential to the intervention, what materials they used, what procedures were involved, and where the intervention was provided. However, most trials failed to describe who provided the intervention, and the quantity of the dose regarding the number of training runs. Additionally, regarding the mental strategy component, RCTs reported generally the type of strategies and strategies instructions but no the specific strategies participants used, we encourage researchers to provide this information in their research.

Additionally, for future systematic reviews we encourage the use of PRISMA statement “http://www.prisma-statement.org (accessed on 11 November 2020)” and a pre-registration of its protocol. During our search for primary studies, we reviewed references for other systematic reviews to search for possible included studies, none of them reported a pre-registered protocol.

Furthermore, more evidence is still needed to establish ways to evaluate the complexity of the intervention, to identify suitable control conditions, and to identify reliable and reproducible neuroimaging biomarkers (more detail of these topics is provided on the discussion section of this systematic review). In addition, when the study hypothesis is based on a biomarker, to find ways to confirm and measure that the biomarker is present in the population at baseline, in that way the modification of a biomarkers behavioral pattern will make more sense instead of only comparing change differences before and after treatment without confirming the biomarker presence.

## 4. Discussion

Real time fMRI neurofeedback training is a developing intervention with potential in neurorehabilitation, as it allows to volitionally regulate brain activity. The overall effect of rt-fMRI-nf on severity depression clinical scores at the end of treatment showed a tendency to favor the intervention, however the global effect was not significant. For the use of rt-fMRI nf training as a neurorehabilitation approach, it is necessary that clinical changes remain after training, yet only 50% of the selected studies reported data at follow-up. From the reported data at follow-up, similar results were found in the overall effect of rt-fMRI-nf and these were not significant. Individually, one study [33,34,35] from the selected trials was able to demonstrate significant differences between groups in severity depression clinical scores at the end of treatment, however not further than one week after follow-up. Quality of life and acceptability were not assessed for most of the selected studies to estimate an effect on these relevant clinical outcomes. Even though randomized control trials are most appropriate to address the beneficial effects of an intervention, a limitation of systematic reviews in general is that the included studies might not be large enough nor long enough to capture relevant data on adverse effects. Only one trial [33,34,35] assessed a cognitive task, which showed an increase in the percent of specific memories recalled and a decrease in the percent of overgeneral memories recalled in the experimental group at follow-up. The clinical relevance of this finding is that individuals with clinical depression recall fewer specific and more categorical autobiographical memories compared with healthy individuals and that this cognitive deficit persists despite remission of symptoms [45,46]; however, more trials are necessary to calculate and give out an estimated effect from RCTs on this specific outcome. Physiology measures other than BOLD activation were not evaluated in any of the selected trials. We found different criteria in the population regarding severity of the depressive disorder at baseline (severe, moderate, and in remission of symptoms). Differences in delivering the intervention as number of feedback training sessions (from one to four nf sessions), feedback modality (two studies assessed activation-based feedback, and two studies assessed connectivity-based neurofeedback), and feedback strategies (positive autobiographical memory recall, imagery of positive stimuli and scenes, negative autobiographical memory recall). As few trials were found, we did not perform a subgroup analysis on these items. 

The mechanism driving clinical effects of neurofeedback are not fully understood; a current debate centers on neurofeedback-specific (related to training a target neurophysiological variable) and non-specific effects. The ‘Consensus on the reporting and experimental design of clinical and cognitive-behavioral neurofeedback studies (CRED-nf checklist) [23], summarizes the publications that focused on the current debate on the mechanisms through which neurofeedback operates; also, it depicted the mechanisms driving experimental outcomes into five bins: neurofeedback-specific (related to training a target neurophysiological variable), neurofeedback non-specific (dependent on the neurofeedback context, but independent from the act of controlling a particular brain signal), general non-specific (including the common benefits of cognitive training as well as psychosocial influences, such as placebo responding), repetition-related (e.g., test–retest improvement), and natural (e.g., spontaneous remission, cognitive development). These mechanisms may interact synergistically to create a greater overall effect, interact antagonistically to reduce the total effect, or combine additively [23]. Regarding neurofeedback-specific (related to training a target neurophysiological variable), all the studies were able to demonstrate changes in brain activity before and after training; however, only one study confirmed success of regulation during a transfer run. These measurements before and after training, were mainly significant in within groups comparisons, in both experimental and control groups. Between groups comparisons might be limited using active control groups. Whole-brain analyses suggests that rt-fMRI-nf alters patterns of activity not only in a single brain region, but brain networks. Evaluation of non-specific effects was not a pre-registered aim of this review; however, we found that some selected studies controlled for or explored some of the non-specific neurofeedback mechanisms that might drive clinical benefits. For example, even though self-efficacy outcome was not included prospectively in our protocol it is worthy of mention that one trial [36] measured self-efficacy using the 1982 version of general and social self-efficacy scales [47] combined. In this trial, the authors found an increase in self-efficacy scores after rt-fMRI-nf training that was associate with less depression severity at the end of treatment. Additionally, cardiorespiratory and motion artifacts are particularly relevant to neurofeedback because participants can inadvertently learn to modify the BOLD signal via artifacts [14], among the selected studies, all use strategies to control for motion artifacts and/or cardiorespiratory artifacts (see Table S5), though no numerical data were reported within or between groups, nor correlations with symptoms improvement were reported. Control conditions are still developing in neurorehabilitation research and variability in control conditions across different clinical trials can introduce substantial clinical heterogeneity [48]. Even though in this systematic review all our included studies were RCTs, the design of a control group itself could cause imbalances if provided different reward experience, motivation, placebo effects, etc. We found variations regarding the type of control groups that the included studies employed. As the reported effect size is estimated from differences between the intervention group and the control group, and none of the included studies assessed all ‘intervention complexity’ factors to evaluate the degree of confounders on the effect estimate, because to evaluate all intervention complexity might be impracticable, we decided to reduce the certainty of the evidence. Additionally, employing neurofeedback control groups, one factor that cannot be controlled for, and that is whether strategy alone would cause the same neural or behavioral changes. In this case, a mental-rehearsal, no feedback, or strategy only control could be apply, but using the mental-rehearsal control condition alone is not sufficient to exclude all possible alternative explanations for an obtained positive behavioral neurofeedback effect, for example, mental rehearsal cannot rule out motivational and placebo effects as this control condition does not include the feedback component, especially the ones related to high-tech environment [49]. Among the selected studies of this systematic review, one study [38], applied mental-rehearsal control while the three others a neurofeedback control group. The results of this study showed no superiority of rt-fMRI-nf training over mental-strategy alone. 

Additionally, to the factors mentioned above, this review identified three main components of the intervention: the region from which nf is provided, direction of regulation, and mental strategy. When intervention complexity is present, it can be difficult to understand which components are most important, and which are responsible for intervention effects (if any), in addition to the interactions between intervention components or interactions between the intervention and its context, or both. Additionally, context may also be influenced by the types and characteristics of participants receiving and delivering the intervention (and their responses), which may subsequently alter the context or the intervention [50].

Furthermore, rt-fMRI-nf training relies on identifying neuroimaging biomarkers that could influence treatment outcome. In this systematic review we identified two studies assessed activation-based feedback, and two studies assessed connectivity-based neurofeedback related to normalize activation patterns. Up to date, several imaging biomarkers of therapeutic response in depression had emerged related to normalize an effect on structural or activation abnormalities; however, before these biomarkers can be translated into clinical practice, they will need to be replicated and validated in large, independent samples, and integrated with data from other biological systems [51]. In addition, since fMRI relies on signal changes that correlates with neural activity, how precisely this hemodynamic response couple or match neural activity is still not fully understood [11,52], and this signal is also dependent on imaging parameters and techniques.

Moreover, a current research drawback on the evaluation of the intervention in depressive disorders and other mental disorders is that the diagnosis is based on the number and type of symptoms, distress, or impairment. This conceptualization makes it difficult for researchers to identify specific aspects of disorders because the neurobiological mechanisms may differ greatly among patients. The diagnosis involves great heterogeneity between patients related with life-span etiology, also everyone with a given diagnosis must possess a minimal number of indicative symptoms, no subset of these must be found in everyone with that diagnosis, and individuals with widely different characteristics can fall within a single diagnostic class and the defining symptom lists for different disorders overlap substantially, so that individuals with different diagnoses can share many symptoms. That is a limitation of the assessment in this systematic review and meta-analysis. To the limitation of scale-based diagnosis, the National Institute of Mental Health (NIMH) Research Domain Criteria (RDoC) emphasize biomarker discovery as a clinical research priority by articulating an approach to the integration of biological and clinical data [24], and future translational neuroscience research could benefit from the RDoC framework to report outcomes with a multidimensional approach. 

Finally, our clinical question was framed for the information provided by the selected trials. Thus, evidence is restricted to only adult patients, the pediatric population was not assessed by any of the selected trials; also, the intervention was narrowed to only visual feedback using a thermometer format and depending on the studies design the thermometer had different colors, and neurofeedback calculations and protocols differ; comparison were heterogenous, and definition of outcomes also differ between studies. We found at least six ongoing trials that we expect might provide additional data in the near future (see Table S3). Some lines of investigation and clinical relevance among these trials are to determine the clinical efficacy of rt-fMRI-nf on augmenting cognitive-behavioral therapy, efficacy of rt-fMRI-nf on treatment-resistant depression, to explore target brain areas and connection patterns, safety, and superiority of the intervention.

The consequences of depressive disorders symptoms in terms of public health are vast [1]. There is still a need to research and develop novel interventions that could enhance treatment effectiveness, account for lower adverse effects, or target resistance-to-treatment populations. We hope that this evidence contributes especially to clinical workers and patients willing to make inform decisions on the current evidence when facing new therapies that might improve clinical symptoms and quality of life of clinical depression. Several factors contribute to evaluate the current evidence from RCTs as still insufficient, and further research efforts may be needed on reveling the mechanism that underly neurofeedback training (specific and non-specific), identifying suitable control conditions, assessing the complexity of the intervention, and finding reliable and reproducible neuroimaging biomarkers, which all together may contribute to elucidate the true effect of rt-fMRI-nf training on patients with clinical depression. 

## 5. Conclusions

The overall effect of rt-fMRI-nf training on improving the severity of depression clinical scores showed in meta-analysis, seems to favor the intervention; however, the overall effect was not significant. Individually, one study from the selected trials, was able to demonstrate significant differences between groups in severity depression clinical scores at the end of treatment, however not further than one week after follow-up. Quality of life and acceptability were not assessed for most of the selected studies to estimate an effect on these outcomes. A limitation of systematic reviews, including this one, is that the included studies might not capture relevant data on adverse effects. All the studies were able to demonstrate changes in brain activity before and after training; however, only one study independently demonstrated regulation success during a transfer run. Whole-brain analyses may suggest that rt-fMRI nf alters activity patterns in brain networks. 

Additionally, it is important to note that our confidence in the effect estimated of relevant clinical outcomes showed in meta-analysis is limited because the certainty of the current evidence from randomized control trials was evaluated as low and very low, and the true effect may be different from the one estimated. We evaluated only RCTs because this study design gathers the best evidence to evaluate interventions. Then, with all the evidence available from RCTs at this moment, we are unable to demonstrate the efficacy of this intervention with certainty for its wide use in clinical settings on the outcomes critical for decision-making in patients with clinical depression. 

## Figures and Tables

**Table 2:** Summary of intervention and match control components.

		Intervention Component		
		Region from Which NF Is Calculated	NF Direction of Regulation	Mental Strategy
Control component	Same as intervention	Zahn et al.	Young et al., Mehler et al.	Young et al., Zahn et al., Jaeckle et al.
	Different from intervention	Young et al., Mehler et al.	Zahn et al.	Mehler et al.
	Do not apply	Jaeckle et al.	Jaeckle et al.	

**Table 3:** Summary of reported outcomes.

	Symptomatology/Disease Severity Reduction	Quality of Life	Acceptability	Adverse Effects	Brain MRI Metrics	Cognitive Tasks	Physiology Measures
Young et al.	✓	x	x	x	✓	✓	x
Mehler et al.	✓	✓	x	✓	✓	x	x
Zahn et al.	✓	x	x	✓	✓	x	x
Jaeckle et al.	✓	x	x	✓	✓ (only intervention group)	x	x

**Table 4:** Summary of Findings (SoF).

Patient or Population: Adults with a Depressive Disorder Intervention: Real-Time fMRI Neurofeedback Training Comparison: Placebo [Active or Non-Active], Another Active Non-Nf Control, or No Treatment				
Outcomes	No. of Participants [Studies]	Certainty of the Evidence [GRADE]	Anticipated Absolute Effects	
			Risk with Control	Risk Difference with Real-Time fMRI Neurofeedback Training
Symptomatology/disease severity reduction at the end of treatment	131 (4 studies)	⨁⨁◯◯ LOW ^a,b,c,d^	-	SMD 0.32 SD lower [0.73 lower to 0.10 higher]
Symptomatology/disease severity reduction at follow-up	61 (2 studies)	⨁◯◯◯ VERY LOW ^a,b,c,d^	-	SMD 0.33 lower [1.91 lower to 1.25 higher]
Increases self-esteem on the Rosenberg global self-esteem scale from baseline to the end of treatment	63 (2 studies)	⨁⨁◯◯ LOW ^a,b,c,d^	-	MD 1.34 higher [0.96 lower to 3.63 higher]
Quality of Life	(1 study)		Not estimable: An estimated effect was not possible, because quality of life was assessed only in one trial. Individually, no significant differences were found between groups.	
Acceptability	(0 studies)		Not estimable: An estimated effect was not possible, because acceptability was not assessed as an outcome in the selected trials.	
Adverse effects [Worsening in symptoms]	(4 studies)	⨁⨁◯◯ LOW ^e^	Low	
			0 per 1000	0 per 1000

Explanations: a. Risk of bias: the certainty of the evidence is downgraded in one level since the overall risk of bias for studies was evaluated as ‘some concerns’ by review authors. Even though most of the items were evaluated as low risk, there were some important concerns regarding the lack of information provided to evaluate some items that we classified as unclear risk, and two studies were evaluated as high risk in performance bias. b. Inconsistency: not serious, serious, or very serious based on the statistical test I^2^ and intervention complexity. The certainty of the evidence is downgraded because of intervention complexity. c. Indirectness: no concerns of indirectness in population. d. Imprecision: the certainty of the evidence is downgraded in one level for imprecision since each end of the confidence interval would lead to different conclusions. e. The analysis of rt-fMRI-nf adverse effects is limited, as we are only examining randomized control trials, also the method of reporting adverse effects was not pre-specified and relies on spontaneous reporting. All of that, in addition with the complexity of the intervention, limit to establish the adverse effect causality.

**Table 5:** Description of regulation success outcomes.

	Explicit Description of Regulation Success by Study Authors	Regulation Outcomes	Regulation Success and Behavioral Correlations
Young et al.	Quote: “Neurofeedback success was defined as the mean percent signal change in the region of interest from the baseline run at visit 2 to the final transfer run at visit 3.”	Both groups successfully regulate activity in the target region. No differences between groups.	Depressive symptoms improved to a greater extent in the experimental group.
Mehler et al.	Quote: “To test whether patients successfully upregulated target areas within a session, ROI analyses were performed for each session. T-values of patient’s target regions were submitted to an ANOVA with the factors intervention (NFE vs. NFS) and session (five levels).”	Both groups regulate activity in the target region. Patients upregulated the target ROIs during all neurofeedback sessions, but not during the transfer session. No differences between groups.	Depressive symptoms improved to a similar extent in both groups.
Zahn et al.	No explicitly described No transfer run or transfer session described Change: [1] increase in correlation between aTL and sACC fMRI signal for guilt on its own. [2] Increase in correlation between aTL and sACC fMRI signal for guilt relative to indignation.” Baseline: before training Comparator: after training	No differences for guilt on its own. Increase in aTL-sACC correlation for guilt vs. indignation in the active vs. control group. However, there was a trendwise decrease in aTL-sACC correlation during the indignation condition after compared to before training in the ACTIVE group and an increase in the CONTROL group.	Positive correlation between self-esteem and connectivity changes, regarding aTL-sACC connectivity increase for guilt vs. indignation.
Jaeckle et al.	No explicitly described No transfer run or session described Change: in the rt-fMRI neurofeedback group: decrease in aTL –posterior SC correlation for self-blame relative to blaming others (using fMRI as measured by regression coefficients for the time series, as extracted by the software FRIEND). Baseline: at the start of visit 2, 1st treatment session. Comparator: at the end of visit 4, last treatment session.	Intervention group successfully regulate. For the control group [mental rehearsal only] this outcome does not apply.	No relationship was found between connectivity changes and the changes in depressive symptoms within intervention group. Self-esteem increased significantly post- vs. pre-treatment [however, in both groups].

**Table 6:** Description of whole brain analyses.

	Explicit Description of Regulation Success BY Study Authors	Regulation Outcomes	Regulation Success and Behavioral Correlations
Young et al.	Quote: “Neurofeedback success was defined as the mean percent signal change in the region of interest from the baseline run at visit 2 to the final transfer run at visit 3.”	Both groups successfully regulate activity in the target region. No differences between groups.	Depressive symptoms improved to a greater extent in the experimental group.
Mehler et al.	Quote: “To test whether patients successfully upregulated target areas within a session, ROI analyses were performed for each session. T-values of patient’s target regions were submitted to an ANOVA with the factors intervention [NFE vs. NFS] and session [five levels].” [Supplementary material]	Both groups regulate activity in the target region. Patients upregulated the target ROIs during all neurofeedback sessions, but not during the transfer session. No differences between groups.	Depressive symptoms improved to a similar extent in both groups.
Zahn et al.	No explicitly described No transfer run or transfer session described Change: (1) Increase in correlation between aTL and sACC fMRI signal for guilt on its own. (2) Increase in correlation between aTL and sACC fMRI signal for guilt relative to indignation.” Baseline: before training Comparator: after training	No differences for guilt on its own. Increase in aTL-sACC correlation for guilt vs. indignation in the active vs control group. However, there was a trendwise decrease in aTL-sACC correlation during the indignation condition after compared to before training in the ACTIVE group and an increase in the CONTROL group.	Positive correlation between self-esteem and connectivity changes, regarding aTL-sACC connectivity increase for guilt vs indignation.
Jaeckle et al.	No explicitly described No transfer run or session described Change: In the rt-fMRI neurofeedback group: decrease in aTL –posterior SC correlation for self-blame relative to blaming others [using fMRI as measured by regression coefficients for the time series, as extracted by the software FRIEND]. Baseline: at the start of visit 2, 1st treatment session. Comparator: at the end of visit 4, last treatment session.	Intervention group successfully regulate. For the control group [mental rehearsal only] this outcome does not apply.	No relationship was found between connectivity changes and the changes in depressive symptoms within intervention group. Self-esteem increased significantly post- vs pre-treatment [however, in both groups].

## Data Availability

Not applicable.

## Supplementary Materials

The following supporting information can be downloaded at: https://www.mdpi.com/article/10.3390/jcm11236909/s1, Table S1: Exclusion criteria of the included studies and baseline characteristics; Table S2a: Summary table explaining the justifications for the allocation of the risk of bias for Young et al; Table S2b: Summary table explaining the justifications for the allocation of the risk of bias for Mehler et al.; Table S2c: Summary table explaining the justifications for the allocation of the risk of bias for Zahn et al.; Table S2d: Summary table explaining the justifications for the allocation of the risk of bias for Jaeckle et al.; Table S3: Ongoing studies and/or results not available; Table S4: Adverse events; Table S5: Control for motion artifacts and/or cardiorespiratory artifacts; Search Strategies for PubMed, Embase.com and CENTRAL.
