# Supplementary material for: Real-Time fMRI Neurofeedback Training as a Neurorehabilitation Approach on Depressive Disorders: A Systematic Review of Randomized Control Trials
Source: J Clin Med. 2022 Nov 23;11(23):6909. doi: 10.3390/jcm11236909 (PMC9737316; doi:10.3390/jcm11236909)
Supplement: Supplementary file 1 [file jcm-11-06909-s001.zip › jcm-2003786-supplementary.pdf]

*Supplementary Material*

**Table S1.** Exclusion criteria of the included studies and baseline characteristics.

| | *Young et al.* | *Mehler et al.* | *Zahn et al.* | *Jaeckle et al.* |
|---|---|---|---|---|
| | | **1. Exclusion criteria** | | |
| General MRI contraindications | ✓ | ✓ | ✓ | ✓ |
| Suicidal ideation/thoughts | ✓ | ? | ✓ | ✓ |
| Current pregnancy | ✓ | ? | ? | ✓ |
| Psychosis | ✓ | ✓ | ? | ? |
| Another major medical condition | Clinically significant or unstable cardiovascular, pulmonary, endocrine, neurological, gastrointestinal illness<br><br>History of traumatic brain injury | ? | ? | History of neurological disorders such as seizures, loss of consciousness following brain injury or medical disorders affecting brain function, blood flow or metabolism |
| Substance abuse | ✓<br>[except for nicotine] | ✓ | ✓ | ✓ |
| Other substances use | Exposure to any medication likely to influence cerebral function or blood flow within 3 weeks.<br>Received psychotropic drugs for at least 3 weeks [8 weeks for fluoxetine] prior to scanning | ? | ? | Current recreational drug use |
| Language restrictions | A primary language other than English | A primary language other than English | A primary language other than Brazilian-Portuguese | A primary language other than English |
| Other than right-handedness | ✓ | No restrictions | ? | ✓ |
| Current pharmacological treatment | ✓ | No exclusions described by authors | No exclusions described by authors | Current intake of benzodiazepines, GABAergic or benzodiazepine receptor agonists |
| Current non-pharmacological treatment | ? | An ongoing non-pharmacological treatment | ? | Currently undergoing psychotherapeutic treatment |
| Other DSM-IV axis-I disorders | No additional DSM-IV axis-I disorders exclusions described by authors | Eating disorders | other current DSM-IV axis-I disorders | History of manic or hypomanic episodes, of schizophreniform symptoms or schizophrenia, or substance abuse<br>Major medical, developmental, or relevant other axis-I disorders |

| | | | | |
|---|---|---|---|---|
| Others | No other exclusions described by authors | No other exclusions described by authors | A history of atypical major depressive episodes [DSM- IV]<br>Prior criminal convictions<br>History of violent behaviour towards persons<br>Positive past or current screening question for irritability on the mood disorders module<br>Antisocial or borderline personality disorder as determined on a personality interview using DSM-IV criteria | ADHD, antisocial or borderline personality disorder<br>Significant impairment of psychosocial functioning before the last MDE indicating the possibility of a comorbid personality disorder<br>Impairments of vision or hearing which cannot be corrected during the treatment sessions<br>History of learning disabilities<br>Past violence or current aggressive impulses |
| | | **1. Baseline characteristics** | | |
| Baseline comorbidities | **Experimental group:**<br>None: 7 [39%]<br>PTSD [post-traumatic stress disorder]: 6 [32%]<br>GAD [generalized anxiety disorder]: 5 [26%]<br>Social Phobia: 3% [16%]<br><br>**Control group:**<br>None: 7 [41%]<br>PTSD [post-traumatic stress disorder]: 3 [18%]<br>GAD [generalized anxiety disorder]: 7 [41%]<br>Social Phobia: 6 [25%] | Not reported | **Experimental group:**<br>Life-time co-morbidity:<br>Bulimia nervosa: 1<br>Anorexia nervosa: 0<br>Panic disorder/agoraphobia: 3<br>Social phobia: 2<br>OCD: 0<br>GAD: 1<br>Specific phobia: 1<br>Health anxiety disorder: 0<br>Multiple anxiety disorders: 1<br>No anxiety disorder: 6<br>Substance abuse: 0<br>Alcohol abuse: 2<br>Alcohol and substance abuse: 1<br>No substance or alcohol abuse: 11<br><br>**Control group:**<br>Life-time co-morbidity:<br>Bulimia nervosa: 0<br>Anorexia nervosa: 1<br>Panic disorder/agoraphobia: 1<br>Social phobia: 0<br>OCD: 1<br>GAD: 0<br>Specific phobia: 3<br>Health anxiety disorder: 1<br>Multiple anxiety disorders: 3<br>No anxiety disorder: 5<br>Substance abuse: 1<br>Alcohol abuse: 1<br>Alcohol and substance abuse: 0 | **Experimental group:**<br>Life-time co-morbidity:<br>Current Persistent Depressive Disorder of the dysthymic subtype: 3<br>Past PTSD with residual symptoms: 3<br>Past PTSD fully remitted: 1<br>Current Social Anxiety Disorder: 2<br>Past Social Anxiety Disorder: 2<br>Past Anorexia Nervosa:1<br><br>**Control group:**<br>Life-time co-morbidity:<br>Current Persistent Depressive Disorder of the dysthymic subtype: 2<br>Past PTSD with residual symptoms: 2<br>Past PTSD fully remitted: 0<br>Current Social Anxiety Disorder: 1<br>Past Social Anxiety Disorder: 0<br>Past Anorexia Nervosa: 0 |

| | | | | |
|---|---|---|---|---|
| | | | No substance or alcohol abuse: 12 | |
| Baseline current medications | No current medication | **Experimental group:** SSRI only = 4; Non-SSRI = 6; Combination = 6 [Combination of two antidepressants = 3; Augmentation = 3; Augmentation included either mood stabilizer, lithium, or 2nd generation antipsychotic in addition to antidepressant]<br><br>**Control group:** SSRI only = 7; Non-SSRI = 5; Combination = 4 [combination of two antidepressants = 0; augmentation = 4; augmentation included either mood stabilizer, lithium, or 2nd generation antipsychotic in addition to antidepressant] | **Experimental group:** SSRI: 6 SNRI: 3 Tricyclic antidepressant:1 [therapeutic dose] Low dose tricyclic antidepressant:1 add-on Topiramate:1 No antidepressant medication: 4 Benzodiazepines: 7 Ritalin: 1<br><br>**Control group:** Current medication SSRI: 8 SNRI: 1 Tricyclic antidepressant:1 [therapeutic dose] Low dose tricyclic antidepressant: 0 add-on Topiramate: 1 No antidepressant medication: 4 Benzodiazepines: 7 Ritalin: 0 | **Experimental group:** Psychotropic medication: 10 Antidepressant [therapeutic dose]: 9 [Of which SSRI:6]<br><br>**Control group:** Psychotropic medication: 10 Antidepressant [therapeutic dose]: 9 [Of which SSRI:6] |
| Baseline socioeconomic status | Not reported | Not reported | Not reported | Not reported |
| Baseline educational level | Not reported | Not reported | **Experimental group:** Years of education: 15.8 [sd:3.4]<br><br>**Control group:** Years of education: 15.0 [sd: 1.9] | **Experimental group:** Years of education: 16.95 [SD: 3.15]<br><br>**Control group:** Years of education mean 18.06 [SD: 2.52] |

**Table S2. a.** Summary table explaining the justifications for the allocation of the risk of bias for Young et al.

| Bias | Authors' judgement | Support for judgement |
|---|---|---|
| Random sequence generation [selection bias] | Unclear risk | Comment: insufficient information about the sequence generation process available to permit a judgement of 'low risk' or 'high risk'. Random sequence generation not described. |
| Allocation concealment [selection bias] | Unclear risk | Comment: insufficient information available to permit a judgement of 'low risk' or 'high risk'. The method of allocation concealment is not described. |
| Blinding of participants and personnel [performance bias] | Low risk | Quote: "Participants and all clinicians and research staff who interacted with participants were blind to assignment." |

| | | |
|---|---|---|
| | | Comment: additionally, the study authors described efforts to maintain blinding during fMRI scanning. The reviews authors judge that blinding of participants and key study personnel are ensured, and unlikely to be broken. |
| Blinding of outcome assessment [detection bias] | Unclear risk | Comment: intention of blinding outcome assessment described on the published protocol; however, it was not described within the published study. |
| Incomplete outcome data [attrition bias] | Low Risk | Comment: attrition rate around to 8% and not power calculation described. However, missing outcome data balanced in numbers across intervention groups, with similar reasons for missing data across groups. |
| Selective reporting [reporting bias] | Low risk | Comment: the study protocol is available and all of the study's prespecified [primary and secondary] outcomes that are of interest in the review have been reported in the prespecified way. |
| Other bias | Unclear risk | Comment: in the control group, memory recall strategy could cause more difficulty in regulate the target region than in the intervention group, causing perhaps difference in reward and motivation. |

**Table S2. b.** Summary table explaining the justifications for the allocation of the risk of bias for Mehler et al.

| Bias | Authors' judgement | Support for judgement |
|---|---|---|
| Random sequence generation [selection bias] | Low risk | Quote: "Patients were randomly assigned to one of two groups using an adaptive randomization protocol developed by the South East Wales Trials Unit [SEWTU]." "The randomization protocol allocated patients to two groups, minimizing for differences in age, gender, duration of illness, medication type... and baseline depression severity... " |
| Allocation concealment [selection bias] | Low risk | Quote: "After the patient had consented and completed all baseline measures, these were entered in a computer program [scripted in Microsoft Excel] ..." |
| Blinding of participants and personnel [performance bias] | High risk | Quote: "Investigators running the MRI sessions needed to know group allocation in order to run the appropriate imaging protocols..." "... the Hamilton Depression Rating Scale ... was administered by a clinician who was blinded to treatment group." "All patients who completed the trial received verbal debriefing at Follow Up." Comment: incomplete blinding, and the outcome was likely to be influenced by lack of blinding. The trial found no group difference in clinical improvement, then it is unlikely that favours intervention. However, it is unknown to which extent this partial blinding could affect the estimate in a positive or negative direction. |
| Blinding of outcome assessment [detection bias] | Low risk | Quote: "... those conducting the assessments were blind to group allocation." Comment: blinding of outcome assessment ensured, and unlikely that the blinding could have been broken. |
| Incomplete outcome data [attrition bias] | Unclear Risk | Quote: "...the attrition rate across both groups was ~26% at the end of the intervention [session 5] and ~35% at follow up." Comment: The power calculation includes a 25% attrition rate. However, reasons for loss to follow-up were not described. |
| Selective reporting [reporting bias] | Low risk | Comment: the study protocol is available and all of the study's prespecified [primary and secondary] outcomes that are of interest in the review have been reported in the prespecified way. |
| Other bias | Unclear risk | Comment: the presentations of scenes in the control group could have had a therapeutic effect itself. |

**Table S2. c.** Summary table explaining the justifications for the allocation of the risk of bias for Zahn et al.

| Bias | Authors' judgement | Support for judgement |
|---|---|---|
| Random sequence generation [selection bias] | Low risk | Quote: "Patients were randomly assigned to one of two groups using an adaptive randomization protocol developed by the South East Wales Trials Unit [SEWTU]." Quote: "The randomization protocol allocated patients to two groups, |

| | | |
|---|---|---|
| | | minimizing for differences in age, gender, duration of illness, medication type... and baseline depression severity... " Comment: the investigators describe a random component in the sequence generation process such as minimization. Differences between groups were found in baseline BDI scores, however not significant. |
| Allocation concealment [selection bias] | Low risk | Comment: participants and investigators enrolling participants could not foresee assignment because a central allocation concealment was used. Quote: "After carrying out the minimised random allocation, he saved the allocation in a text file which he uploaded onto the FRIEND server directly, thereby assuring concealment from the research team." |
| Blinding of participants and personnel [performance bias] | Low risk | Quote: "Researchers were unblinded only after completing all assessments by looking at the text file that indicated the group allocation. Participants were not unblinded." Comment: blinding of participants and key study personnel ensured, and unlikely that the blinding could have been broken. |
| Blinding of outcome assessment [detection bias] | Low risk | Comment: blinding of outcome assessment ensured, and unlikely that the blinding could have been broken. Quote: "Researchers were unblinded only after completing all assessments by looking at the text file that indicated the group allocation." |
| Incomplete outcome data [attrition bias] | Unclear Risk | Comment: even though the study state in general the initial and final number of participants taking symptomatology measures, it does not specify to which group correspond the ~14% participants who did not complete all questionnaires, in order to evaluate if the losses are balanced. |
| Selective reporting [reporting bias] | Low risk | Comment: the study protocol is available. Unplanned exploratory outcomes were additionally included, but all the study's prespecified [primary and secondary] outcomes that are of interest in the review have been reported in the prespecified way. |
| Other bias | Unclear risk | Quote: "the ACTIVE intervention appears to have been more difficult and thus patients had a much lower thermometer level display on average, thus getting much less positive feedback on their performance than the CONTROL intervention group." |

**Table S2. d.** Summary table explaining the justifications for the allocation of the risk of bias for Jaeckle et al.

| Bias | Authors' judgement | Support for judgement |
|---|---|---|
| Random sequence generation [selection bias] | Low risk | Comment: the investigators describe a random component in the sequence generation process such as stratification performed by an automatised online system. Quote: "The randomisation of trial participants was performed by an automatised online system, set up by the Clinical Trials Unit, King's College London. The randomisation process implied a stratified block design with randomly varying block sizes, deploying two stratification factors: gender [female/male] and baseline scores of the primary outcome measure, the Beck Depression Inventory-II..." |
| Allocation concealment [selection bias] | Unclear risk | Comment: insufficient information available to permit a judgement of 'low risk' or 'high risk'. The method of allocation concealment is not described. |
| Blinding of participants and personnel [performance bias] | High risk | Quote: "Participants were informed about their allocated treatment group upon completion of the baseline clinical and neuropsychological testing on their pre-treatment assessment [visit 1]." Comment: no blinding, and the outcome was likely to be influenced by lack of blinding. |
| Blinding of outcome assessment [detection bias] | Low risk | Quote: "Observer-rated outcomes were assessed by a senior psychiatrist [R.Z. or A.C. in his absence] who was blinded to the treatment group allocation of participants throughout the trial." |

| | | |
|---|---|---|
| Incomplete outcome data [attrition bias] | Low Risk | Comment: power calculation considered a 20% drop out rate, which was no surpassed. Missing outcome data is balanced in numbers across intervention groups. |
| Selective reporting [reporting bias] | Low risk | Comment: the study protocol is available and all of the study's prespecified [primary and secondary] outcomes that are of interest in the review have been reported. |
| Other bias | Unclear risk | Comment: participants in the control group are placed not in the same high-tech MRI environment [outside the scanner]. |

**Table S3.** Ongoing studies and/or results not available.

| | Ongoing studies and/or results not available | Trial Register Number | Summary | Preliminary results |
|---|---|---|---|---|
| *1.* | Effects of Amygdala Neurofeedback on Depressive Symptoms.<br><br>[Responsible Party: Kymberly Young, Assistant Professor of Psychiatry, University of Pittsburgh] | NCT02709161 | "The purpose of this study is to determine the clinical efficacy of augmenting cognitive-behavioural therapy with real-time functional magnetic resonance imaging neurofeedback [rtfMRI-nf] training to increase the amygdala's response to positive autobiographical memories." | Not found |
| *2.* | Neurofeedback for Treatment Resistant Depression.<br><br>[Responsible Party: Kymberly Young, Assistant Professor of Psychiatry, University of Pittsburgh] | NCT03428828 | "The purpose of this study is to determine the clinical efficacy of real-time functional magnetic resonance imaging neurofeedback [rtfMRI-nf] training to increase the amygdala's response to positive autobiographical memories in patients with depression who are considered treatment-resistant." | Not found |
| 3. | Real-time Biofeedback With 7-Tesla MRI for Treatment of Depression.<br><br>[Responsible Party: Laurel Morris, Postdoctoral Research Fellow, Icahn School of Medicine at Mount Sinai] | NCT04138680 | "Our question is whether VTA self-modulation with biofeedback can influence depression symptoms." | Not found |
| *4.* | Can neurofeedback-based modification of functional cerebral asymmetries and anterior cingulate activation improve the treatment of depressive disorders?<br><br>[Primary Sponsor: Institut für klinische Radiologie - TRICGebäude A 16 Universitätsklinikum Münster/ Dr. Susanne Bergert] | DRKS00012261 | "Depressive disorders typically go along with several characteristic neuronal features. Among other things, there is a dysbalance of the activations within the prefrontal cortex, a brain structure dedicated to executive functions, working memory and decision making. While the left prefronal cortex is usually too weakly activated, the right prefronal cortex is often too strongly activated. In this study, we therefore attempt to reduce this dysbalance in order to see how much depressive symptoms abate in the process.<br>In addition, depressive patients often show a hyperconnectivity within the prefrontal cortex and possibly also a hyperconnectivity between prefrontal cortex and amygdala. Since previous studies demonstrated that MRI-based neurofeedback cannot only alter the activity of particular brain regions, but also influence the | Not found |

| | | | | |
|---|---|---|---|---|
| | | | connections between brain structures, we want to observe this aspect too.” | |
| *5.* | A semi-crossover open trial of combined therapy of fMRI neurofeedback and repetitive transcranial magnetic stimulation [rTMS] for affective disorders.<br><br>[Principal investigator: Hidehiko Takahashi, Department of Psychiatry, Kyoto University Graduate School of Medicine]. | JPRN-UMIN000013272 | “A main purpose of this clinical trial is to evaluate safety and efficacy, including antidepressant effect and prefrontal cognitive enhancement, of fMRI neurofeedback and rTMS applied to major depressive episode.” | Not found |
| *6.* | A real-time fmri neurofeedback for mild to severe depression compared to frontal alpha-asymmetry neurofeedback and cognitive-behavioural therapy<br>[Author: Mikhail Melnikov, Federal Research Center of Fundamental and Translational Medicine, Novosibirsk, Novosibirskaya Oblast', Russia]. | Not found | “The aim of our study was to examine effects of the real-time fMRI neurofeedback as a treatment arm for mild to severe depression. Alpha-asymmetry neurofeedback and cognitive-behavioural therapy [CBT] served as control treatment arms.” | [Abstract conference] Results: Patients from all the groups significantly improved from the treatment. A status of some patients according to DSM-5 changed to milder depression or to no depression condition. The fMRI- neurofeedback group showed significant improvements on MADRS, BDI, SDS, and HADS that were statistically comparable with those in alpha- asymmetry neurofeedback and CBT. Patients of the fMRI group demonstrated ability to control prefrontal cortex signal both in usual feedback and in transfer [no feedback] sessions and gained positive changes of emotional state during sessions. |

**Table S4.** Adverse events.

| Event type - category | Description | Total number of participants presenting adverse events | | Numbers of withdrawals due to adverse events. | |
|---|---|---|---|---|---|
| | | Experimental group | Control Group | Experimental group | Control Group |
| Severity of depression | Worsening in symptoms | 2 | | | 1* |
| During or after rehearsals, practice, or homework | Frustration, mainly because of difficulty focusing and concentrating | 3 | 3 | | 0 |
| During MRI scanning | Physical discomfort during scanning, or participant move excessively during scanning | 2 | 2 | 2 | 2 |
| After MRI scanning | Exhaustion or fatigue after scanning | 1 | 5 | | |
| Headaches or dizziness or other aches | Headaches or dizziness or other aches during or after scanning | 2 | 3 | | |
| Acoustic | Transient ear ringing after one scanning session. | 1 | | | |
| Insomnia | Insomnia after NF session | | 1 | | |
| Agitation | Agitation after scanning | 1 | | | |

*Symptoms of depression worsened by 10 points on the BDI-II between baseline assessment and first intervention day, participant did not received NF as it belongs to control group. (Jaeckle et al.)

**Table S5.** Control for motion artifacts and/or cardiorespiratory artifacts.

| | Measurement tool |
|---|---|
| Young et al. | "Pre-processing of single-subject fMRI data included correction of cardiorespiratory artifacts using AFNI implementation of the RETRICOLOR method." |
| Mehler et al. | "To control for physiological confounding factors of the BOLD signal, heart rate [HR] and respiration volume per time [RVT] were measured using pulse oximetry and a respiratory belt, respectively, and recorded with Spike2 [version 5.21, Cambridge Electronics Design Limited, Cambridge, UK]." |
| Zahn et al. | "Root mean squares [RMS] of movement parameters for translation and rotation were tracked on an ongoing basis and the neurofeedback screen displayed a warning to participants and investigators in real- time if movement exceeded allowable levels, excluding those volumes from calculations of feedback signal [see Supplementary Methods]. Unfortunately, FRIEND does not record the number of these excluded volumes during real-time neurofeedback." |
| Jaeckle et al. | "While being in the MRI scanner, the participant's head motion was restricted using padding and heart rate measurements recorded via a finger pulse sensor." |

## Search Strategy

*PubMed*

(((mood* OR affective*) AND (disorder* OR illness*)) OR dysthymi* OR depressi* OR unipolar* OR MDD OR ((premenstrual OR menstrual* OR "pre-menstrual") AND dysphori*) OR PMDD) AND ((“real-time functional MRI” OR “rt-fMRI-nf” OR “rtfMRI-nf” OR “functional-MRI” OR fMRI* OR rtfMRI* OR (functional* AND ("magnetic resonance" OR MRI*) AND (real-time OR “real time”)) OR ((BCI* OR BMI* OR “brain-computer” OR “brain-machine” OR “direct neural” OR (brain AND (computer* OR machine* OR interfac* OR regulat* OR “self-regulation”))))) AND (neurofeedback* OR "neuro feedback" OR “neuro-feedback” OR feedback))

*Embase.com*

(((mood* OR affective*) AND (disorder* OR illness*) OR dysthymi* OR depressi* OR unipolar* OR mdd OR ((premenstrual OR menstrual* OR 'pre-menstrual') AND dysphori*) OR pmdd)) AND ('real-time functional mri' OR 'rt-fmri-nf' OR 'rtfmri-nf' OR 'functional-mri' OR fmri* OR rtfmri* OR (functional* AND ('magnetic resonance' OR mri*) AND 'real time') OR bci* OR bmi* OR 'brain-computer' OR 'brain- machine' OR 'direct neural' OR (brain AND (computer* OR machine* OR interfac* OR regulat* OR 'self- regulation'))) AND (neurofeedback* OR 'neuro feedback' OR 'neuro-feedback' OR feedback) AND ([controlled clinical trial]/lim OR [randomized controlled trial]/lim)

*CENTRAL*

(((mood* OR affective*) AND (disorder* OR illness*)) OR dysthymi* OR depressi* OR unipolar* OR MDD OR ((premenstrual OR menstrual* OR "pre-menstrual") AND dysphori*) OR PMDD) AND ((“real- time functional MRI” OR “rt-fMRI-nf” OR “rtfMRI-nf” OR “functional-MRI” OR fMRI* OR rtfMRI* OR (functional* AND ("magnetic resonance" OR MRI*) AND (real-time OR “real time”)) OR ((BCI* OR BMI* OR “brain-computer” OR “brain-machine” OR “direct neural” OR (brain AND (computer* OR machine* OR interfac* OR regulat* OR “self-regulation”))))) AND (neurofeedback* OR "neuro feedback" OR “neuro-feedback” OR feedback))
